# APSIC guidelines for environmental hygiene: surface cleaning air and water quality in hospitals: 2025 update

**DOI:** 10.1017/ash.2025.10288

**Published:** 2026-01-28

**Authors:** Anucha Apisarnthanarak, Moi Lin Ling, Namita Jaggi, Patricia Ching, Lily Liang, Zhiyong Zong

**Affiliations:** 1 Division of Infectious Diseases, Thammasat University Hospital, Pathum Thani, Thailand; 2 Infection Prevention & Epidemiology, Singapore General Hospital, Singapore; 3 Labs & Infection Control and Chief Education & Research, Artemis Hospital, Delhi, India; 4 WHO Collaborating Centre for Infectious Disease Epidemiology and Control, School of Public Health, The University of Hong Kong, China, Hong Kong; 5 Dental Services, National Healthcare Polyclinics, Singapore; 6 Center of Infectious Diseases, West China Hospital, Sichuan University, Sichuan, China

## Abstract

**Objective:**

To describe the revised APSIC Environmental Hygiene Guidelines for prevention of healthcare-associated infections inclusive of surface cleaning, air and water quality.

**Design:**

The revised guideline was developed by Infection Prevention and Control key opinion leaders from Asia Pacific.

**Setting:**

This guideline emphasizes on practical implementation of environmental hygiene for prevention of healthcare-associated infections inclusive of surface cleaning, air and water quality relevant to Asia Pacific settings.

**Patients or participants:**

Any patients hospitalized in healthcare setting.

**Interventions:**

Literature search was done for recent international updates in environmental hygiene inclusive of surface cleaning, air and water quality. Recommendations were evaluated for practical and feasible recommendation in low-resourced settings in Asia Pacific.

**Results:**

The key recommendations are listed in the best practices for cleaning patient care areas. Additional measures are recommended to improve the air and water quality in healthcare settings.

**Conclusions:**

Implementation of environmental hygiene in Asia Pacific should take into consideration of the air and water quality in addition of surface cleaning. Measures to assess the cleanliness should be performed using conventional visual assessment, environmental cleaning and disinfection checklist, auditing, and additional measures (e.g., environmental culture or fluorescence).

## Introduction

Contamination of hospital surface, air, and water supplies with hospital pathogens is a well-recognized associated cause of common-source outbreaks and healthcare-associated infections (HAIs).^1–2^ Hospitalized patients shed pathogens into their surrounding environments that may resulting in surface contamination which can serve as a source for subsequent transmission.^1–2^ In recent years, air and water quality also serve as an important source of pathogens transmission.^3–27^ Pathogen transfer from an affected patient to a susceptible host occurs most commonly via the hands of healthcare personnel (HCP), but contaminated objects, surfaces, water and air can be either directly or indirectly involved in the transmission pathway.^1–27^ Therefore, new healthcare facility design and hygienic practices have been largely directed at controlling healthcare-associated pathogens that come from contaminating surfaces, hands, equipment, water and air.

Contaminated surfaces, water and air quality make an important contribution to the epidemic and endemic transmission of *Clostridioides difficile*, vancomycin-resistant enterococci (VRE), methicillin-resistant *Staphylococcus aureus* (MRSA), *Acinetobacter baumannii*, *Pseudomonas aeruginosa, Candida auris* and that improved environmental decontamination contributes to the control of outbreaks bacterial and fungal spores, and viruses that are shed from infected and/or colonized patients (and sometimes HCP) into the hospital environment.^2–27,4–5^ Surfaces in the vicinity of patients that are touched frequently by HCP and patients—termed “high-touch surfaces” (defined by direct observation)—have a higher frequency of contamination than other sites. In addition, healthcare-associated pathogens can survive on surfaces for prolong period.^6^ A number of studies have also identified the previous presence of a colonized or infected patient in a side room as a risk factor for the acquisition of the same pathogen by a new occupant.^7^ This effect has been shown for VRE, MRSA, *C. difficile*, multidrug-resistant *P. aeruginosa*, and *A. baumannii*.^5,7^ Evidence in Asia Pacific suggest that the contaminated surface environment contributes to the transmission of healthcare-associated pathogens and that improved cleaning and disinfection reduces the risk of HAIs.^8–9^ In this guideline, we provide evidence and practices of environmental hygiene focusing on new recommendation on surface cleaning, air and water quality issues that relevant to practices in Asia Pacific (full guideline available at: https://apsic-apac.org).

## Methods

The workgroup reviewed previously published relevant international guidelines and recommendations and performed literature searches using PubMed. The workgroup met twice on online platform to discuss all contents in the guidelines as well as discussed via email correspondences to complete the revision of the guidelines. Criteria used for grading the strength of recommendations and quality of evidence are described in Table 1.

**Table 1.** Categories for recommendation

Categories for strength of each recommendation	
Category	Definition
A	Good evidence to support a recommendation for use.
B	Moderate evidence to support a recommendation for use.
C	Insufficient evidence to support a recommendation for or against use.
D	Moderate evidence to support a recommendation against use.
E	Good evidence to support a recommendation against use.

Categories for quality of evidence on which recommendations are made	
Grade	Definition
I	Evidence from at least one properly randomized, controlled trial.
II	Evidence from at least one well-designed clinical trial without randomization, from cohort or case-controlled analytic studies, preferably from more than one center, from multiple time series, or from dramatic results in uncontrolled experiments.
III	Evidence from opinions of respected authorities on the basis of clinical experience, descriptive studies, or reports of expert committees.

The draft guideline was sent to an external reviewer, APSIC executive committee and national infection prevention and control societies who are member societies of APSIC in Asia Pacific for comments and feedback. Final draft was then made following revision made according to comments and feedback received. It was endorsed by the APSIC executive committee before upload at the APSIC website.

## Results

### General cleaning practices

Health care settings comprised areas that require either hotel clean or hospital clean based on the risk of the patient population in the area. Hotel Clean is a measure of cleanliness based on visual appearance that includes dust and dirt removal, waste disposal and cleaning of windows and surfaces. In addition to routine cleaning, additional cleaning practices and/or the use of personal protective equipment for cleaning may be required in health care settings under special circumstances. Hotel Clean is the basic cleaning that takes place in all areas of a health care setting.^10^ Hospital Clean is a measure of cleanliness routinely maintained in care areas of the health care setting. Hospital Clean is “*Hotel Clean”* with the addition of disinfection, increased frequency of cleaning, auditing, and other infection control measures in client/patient/resident care areas.^10^ It is highly recommended that Infection Prevention and Control (IPC), Occupational Health and Safety, and Environmental Service collectively in decision making with respect to choices of furniture and finishing for the facilities.

### Components of hotel clean

- Floors and baseboards are free of stains, visible dust, spills, and streaks
- Walls, ceilings, and doors are free of visible dust, gross soil, streaks, spider webs, and handprints
- All horizontal surfaces are free of visible dust or streaks (includes furniture, window ledges, overhead lights, phones, picture frames, carpets, etc.)
- Bathroom fixtures including toilets, sinks, tubs, and showers are free of streaks, soil, stains, and soap scum
- Mirrors and windows are free of dust and streaks
- Dispensers are free of dust, soiling, and residue and replaced/replenished when empty
- Appliances are free of dust, soiling, and stains
- Waste is disposed of appropriately
- Items that are broken, torn, cracked, or malfunctioning are replaced

### *Cleaning best practices at patient care areas* ^10–14^

Environmental Services (EVS) in the health care setting should be performed on a routine and consistent basis to provide for a safe and sanitary environment. Maintaining a clean and safe health care environment is an important component of infection prevention and control. The frequency of cleaning and disinfecting individual items or surfaces in a particular area or department depends on (Table 2):

- Whether surfaces are high-touch or low-touch;
- The type of activity taking place in the area and the risk of infection associated with it (e.g., critical care areas vs meeting room);
- The vulnerability of patients housed in the area; and
- The probability of contamination based on the amount of body fluid contamination surfaces in the area might have or be expected to have

**Table 2.** Example of risk stratification matrix to determine the frequency of cleaning

Units	Probability of contamination^a^ Light 1 Moderate 2 Heavy 3	Potential for exposure^b^ Hi-touch 3 Low-touch 1	Population Less susceptible-0 More susceptible-1	Total Score^c^
Dialysis unit	3	3	1	7
Intensive care unit	3	3	1	7
Triage area	1	1	0	2

Note. ^a^Heavy contamination: surfaces and/or equipment that are routinely exposed to copious amount of blood and/or other body fluids (e.g., burn unit, dialysis unit, emergency department). Moderate contamination: surfaces and/or equipment are not routinely, but may become contaminated with blood and/or other body fluids (e.g., patient room, bath room if patient is incontinent). Light contamination: surfaces and/or equipment that are not expose to blood or body fluid. Interpretation of total score (e.g., lounges, office).

b Hi-touch: surfaces that have frequent contact with hand (e.g., door knobs, bed rails, computer keyboard); Low-touch: surfaces that have minimal contact with hands (e.g., wall, ceiling, mirror).

c 7: High risk: clean after each case/event/procedure and at least twice per day, clean additionally as required. 4–6: Moderate risk: clean at least once daily, clean additionally as required (e.g., gross soiling). 2–3: Low risk: clean according to a fixed scheduled, clean additionally as required (e.g., gross soiling).

### Recommendations

1. EVS in the health care setting should be performed on a routine and consistent basis to provide for a safe and sanitary environment. [BIII]
2. Adequate resources must be devoted to EVS Department in all health care settings that include:
  - Single individual with assigned responsibility for the care of the physical facility;
  - Written procedures for cleaning and disinfection of care areas and equipment that include:
    - Defined responsibility for specific items and areas;
    - Procedures for daily and terminal cleaning;
    - Procedures for cleaning in construction/renovation areas;
    - Procedures for cleaning and disinfecting areas contaminated with VRE and *C. difficile*;
    - Procedures for outbreak management;
    - Cleaning standards and frequency;
  - Adequate human resources to allow thorough and timely cleaning and disinfection;
  - Education and continuing education of cleaning staff;
  - Monitoring of environmental cleanliness; and
  - Ongoing review of procedures. [BII]
3. If EVSs are contracted out, the Occupational Health and Safety policies of the contracting services must be consistent with the facility’s Occupational Health and Safety policies. [BII]
4. EVS Department staffing levels should reflect the physical nature and the acuity of the facility; levels of supervisory staff should be appropriate to the number of staff involved in cleaning. [BIII]
5. Non-critical medical equipment requires cleaning and disinfection after each use. [AII]
6. Each health care setting should have written policies and procedures for the appropriate cleaning of non-critical medical equipment that clearly defines the frequency and level of cleaning and which assigns responsibility for the cleaning. [BIII]
7. Cleaning schedules should be developed, with frequency of cleaning reflecting whether surfaces are high-touch or low-touch, the type of activity taking place in the area and the infection risk associated with it, the vulnerability of the patients housed in the area, and the probability of contamination. [BIII]
8. All environmental service staff entering a room, which is on contact precautions, must put on a gown and gloves on entering the room and must remove them and perform hand hygiene on leaving the room. [BII]
9. For adequate removal of *C. difficile*, the use of a sporicidal agent for disinfection after the room has been cleaned is needed. [BII]
10. Environmental cleaning and disinfection of a room of a patient with *C. auris* should be performed using EPA-registered or healthcare disinfectant with claims of efficacy against *C. auris*. Sodium hypochlorite (bleach) at 1,000 ppm is effective, especially for terminal cleaning, but follow dilution and usage instructions carefully. [BII]
11. EVS staff entering a room with airborne precautions must wear a fit-tested N95 respirator. [BII]
12. EVS and clinical staff entering a room with droplet precautions must wear a surgical mask. [BII]
13. IPC, Environmental services, and Occupational Health and Safety should be involved in the selection of surfaces and finishes in healthcare settings **[CII]**.
14. Surfaces, furnishings, equipment, and finishes in health care settings should [BII]:
  - Be easily maintained and repaired;
  - Be cleanable with hospital-grade detergents, cleaners, and disinfectants;
  - Be smooth, nonporous, seamless, and unable to support microbial viability.
15. Carpets should not be used in patient care areas and especially in areas that house or serve immunocompromised patients or where there is a high likelihood of contamination with blood or body fluids. **[BII]**

### Cleaning agent and non-touch technologies

The ideal properties of disinfectants should include their nature of broad-spectrum, rapid action, persistency, material compatibilities, non-flammable. Several common disinfectants that commonly used in the healthcare include quaternary ammonium compounds, alcohols (ethyl or isopropyl), chlorine releasing agents (eg, bleach), improved hydrogen peroxide. Several non-touch’ technologies such as hydrogen peroxide vapor systems, ultraviolet-C light devices and electrostatic spraying are supplementary options for terminal disinfection and outbreak situations. Their advantage is they eliminate human factors such as relying on an operator to ensure that all surfaces are disinfected adequately. Multiple studies in Asia Pacific have proven their efficacy during terminal cleaning (PX-UV) and during flood (hydrogen peroxide).^15–18^ However, the convenience of using these technologies for routine cleaning and disinfection is limited due to cost considerations, regulatory requirements, and the need for prior conservative wipe disinfection.

### Recommendations

1. Cleaning product should be approved by occupational health and safety and environmental services and must be used according to the manufacturers’ recommendations (e.g., material compatibility, storage, shelf life, and safe use). **[CIII]**
2. Surface cleaning cloths and mop heads or floor cloths should be cotton or microfiber; the frequent laundering of cotton-string mops (e.g., daily) is recommended. **[CIII]**
3. “No-touch” technologies such as hydrogen peroxide vapor systems and ultraviolet-C light devices may be considered for terminal room decontamination, especially if the patient had a high-consequence pathogen (e.g., Ebola, *C. auris*). **[BII]**

### Cleaning in special settings

#### Dental offices

The dental offices are classified as clinical contact and environmental surfaces. Clinical contact surfaces are considered high-touch surfaces.^19^ During dental procedures it generate a lot of aerosols and spatters which is heavily contaminated by microorganisms from the mouth and dental unit waterline.

#### Recommendation

1. Use water that meets the United States Centres for Diseases Control and Prevention (CDC) recommended limit for dental procedural water (i.e., <500 CFU/mL of heterotrophic water bacteria) for routine dental treatment [AII].
2. Every dental unit waterline should be treated regularly with appropriate disinfectants to meet regulatory standards [CI]
3. Dental unit water quality must be monitored, or tested, routinely as recommended by the equipment manufacturer [BI].
4. When bacterial levels in Dental Unit Waterlines (DUWLs) exceed 500 CFUs, potential next steps for practice may include shocking and/or treating the waterline, along with additional testing [BI].
5. Unused waterlines, often referred to as dead legs should be properly and effectively terminated [BII].
6. Monitoring dental unit water quality help identify performance problems or compliance with maintenance procedures and provide documentation of compliance.
7. Ensure flushing of DUWLs for 20–30 seconds before the start of the day and between patients [BII].

#### Ambulance

Both surfaces and air are contaminated by organisms commonly associated with healthcare-associated infections in the ambulances such as MRSA, VRE, and carbapenem-resistant *A. baumannii*, carbapenem-resistant *P. aeruginosa, and C. auris*.^20^ Blood pressure cuff, oxygen apparatus, stretcher, and patient compartment area are the most common contaminated site.

#### Recommendation

1. High-touch surfaces including the stretcher, the blood pressure cuff, the oxygen knob, and the doorknob should be wiped with detergent or bleach after every patient transportation as soon as possible. [AIII]
2. Thorough cleaning should be performed once a day, including cleaning the patient area, wiping all surfaces, and sweeping the floor. [AIII]
3. Enhanced cleaning should be performed after the transportation of each patient with any of the following: diarrhea or vomiting, urinary incontinence, communicable diseases transmitted through the air, emerging infectious diseases, diseases of an outbreak or a cluster comprising multiple cases, multi-drug resistant organisms (carbapenem-resistant Gram-negative organisms, MRSA, or VRE), *Candida auris*, and any other conditions with the potential to contaminate the ambulance and cause transmission. [AIII]

### Assessment of cleanliness and quality

Methods for assessing environmental cleanliness can be performed using conventional program of direct and indirect observation (eg, visual assessment, observation of performance) and enhanced program of monitoring residual bioburden (eg, environmental culture, adenosine triphosphate—ATP—bioluminescence); and environmental marking tools (eg, fluorescent marking). In a Thai national survey on environmental cleaning and disinfection,^21^ implementing environmental cleaning and disinfection protocol was commonly performed in majority of Thai hospitals (90%), but assessment of cleanliness was less commonly performed (45%). Notable, Hospital epidemiologist presence was associated with the existence of an environmental cleaning and disinfection (ECD) checklist and of ECD auditing, while good and excellent hospital administrative support were associated with better adherence to ECD protocols and ECD checklists. The data emphasize the role of administration support, presence of hospital epidemiologist to be important prerequisite for effective monitoring of cleanliness and quality control.

### Recommendation

1. There should be a process in place to measure the quality of cleaning in the healthcare setting. [AII]
2. Methods of monitoring cleanliness should include at least the conventional visual assessment and/or fluorescent marking. [AII]
3. Results of cleaning audits should be collated and analyzed with immediate feedback to staff, and an action plan developed to identify and correct deficiencies. [AII]
4. Result of fluorescent monitoring should be reported the IPC Committee periodically (e.g., monthly). [AII]

### Infection control during construction and renovation

1. **Infection prevention and control during construction and renovation**

Construction and renovation activities in the hospitals may be associated with transmission of pathogens such as filamentous fungi, including *Aspergillus spp*, *Candida spp*, *Fusarium* and also bacteria such as *Legionella* and *Nocardia*.^22–24^ Hospital construction-related infection such as Aspergillosis represents the greatest threat to neutropenic and immunocompromised patients. Prior to the construction and renovation activities, an “Infection Control (IC) Risk Assessment” (Appendix A) must be completed. The risk assessment consists of the following 3 steps:-

1. Identify the type of construction project. (Step 1)
2. Identify those patient areas at risk. (Step 2)
3. Match the type of construction activity with the patient risk group. (Step 3)

Infection control precautions to be taken for respective class of risks are described in Appendix A.

#### Recommendations

1. Prior to any construction or renovation activity, patients who are at risk should be identified as high-risk, medium-risk and low-risk patients. [BIII]
2. Preconstruction and renovation consultation should be carried out in advance between all the stakeholders. [BIII]
3. During construction activities, it is necessary to contain or minimize dispersal of dust. [BIII]
4. Once the project is started, the Infection Control Team shall conduct rounds in order to verify infection control compliance. [BIII]
5. If corrective measures are not adequate, the Head of Department of Infection Control has the authority to stop further work on the renovation/construction project until corrective measures are adequately addressed. [CIII]

### Air and water quality

#### Indoor air quality and ventilation system

Respiratory viruses may be transmitted through fine droplets or droplet nuclei especially when aerosol generating procedures (AGPs) are done in clinical areas.^25–26^ Hence, there should be adequate ventilation within each setting to prevent possibility of transmission during routine patient care activities. The general purpose of ventilation in buildings is to provide healthy air through dilution and removal of existing pollutants taken into account of ventilation rate, airflow direction, air distribution, or airflow plan. Building may be ventilated using natural ventilation, mechanical ventilation or hybrid/mixed-mode ventilation. Critical ventilation systems should be inspected at least 6 monthly and certified at least once every two years. Carbon dioxide (CO_2_) monitoring may be used as a proxy for ventilation adequacy where the recommendation is keeping CO_2_ below 800 ppm.

In assessing ventilation performance, the key questions posed are:

1. Does the system provide sufficient ventilation rate (i.e., air changes per hour (ACH)) or flow rate in L/sec/person?
  - Minimum 160 L/sec/person or 12 ACH where AGPs are performed.
  - Minimum 60 L/sec/person or 6 ACH for general wards and outpatient departments.
  - Minimum 2.5 L/sec/m^3^ for corridors and other transient spaces without fixed patient numbers.
2. Is the airflow direction from a clean to less clean zone?
3. Is air quality adequate for immunocompromised patients (e.g., stem cell transplants)?
  - Ideally, these should be housed in appropriate rooms with HEPA-filtered units and positive pressured.

#### Water quality

Wet environments pose some risks for healthcare-associated infections in the healthcare setting and promotes microbial growth and serve as a source for antimicrobial-resistant pathogens. Certain conditions within the plumbing system (eg, sink and drains, shower heads, etc.) may encourage microbial growth or biofilm development.^27^ A healthcare water management program is highly recommended to identify risk points and corrective actions planned to minimize the growth and spread of waterborne pathogens.^27^ The healthcare facility should have a water management policy that addresses the following:

- Surveillance or monitoring system for *Legionella* growth at cooling towers, shower head, and corrective actions to be taken in response systems performing outside of control limits.
- Response plan in event of identification of a patient with healthcare-onset Legionellosis and/or outbreaks [e.g., ≥1 case of definite healthcare-associated legionellosis in patient who has been admitted to unit at least 10 days prior to onset of illness or ≥2 case of possible healthcare-associated legionellosis identified within 12 months apart].

The water management program should be reviewed regularly and revised accordingly when any of the following events occur:

- Data review (e.g. shower heads sampled for *Legionella* culture)—results that suggests that control measures are persistently out of control limits
- Major maintenance or water service change e.g. new construction, changes in municipal water supply, changes in equipment or treatment products (Appendix B, C).

#### Recommendations

1. The IPC team should be in close collaboration with the architects, engineers and project teams in designing of new facilities or renovation of existing healthcare facilities. **[AIII]**
2. Critical ventilation systems should be inspected at least 6 monthly and certified at least once every two years or as required by national requirement. **[BIII]**
3. A healthcare water management program is recommended to identify risk points and corrective actions planned to minimize the growth and spread of waterborne pathogens. **[AIII]**

## Supporting information

10.1017/ash.2025.10288.sm001 Apisarnthanarak et al. supplementary material Apisarnthanarak et al. supplementary material

## Supplementary material

The supplementary material for this article can be found at https://doi.org/10.1017/ash.2025.10288.
