# Supplementary material for: APSIC guidelines for environmental hygiene: surface cleaning air and water quality in hospitals: 2025 update
Source: Antimicrob Steward Healthc Epidemiol. 2026 Jan 28;6(1):e34. doi: 10.1017/ash.2025.10288 (PMC12854877; doi:10.1017/ash.2025.10288)
Supplement: Apisarnthanarak et al. supplementary material [file S2732494X2510288Xsup001.docx]

**Appendix A:**

**STEP 1: Identify the Infection Control Risk Assessment (ICRA)**

**TYPE OF CONSTRUCTION ACTIVITY OR PROJECT (circle type of project):**

| **Type A** | **Inspection and non-Invasive Activities**  Includes but not limited to:   - Activities which do not generate dust or require cutting of walls or access to ceilings other than for visual inspection.   e.g., Removal of ceiling tiles for visual inspection, painting but not sanding, electrical work, minor plumbing that disrupt water supply to localized patient care area (e.g. in one room) |
| --- | --- |
| **Type B** | **Small scale short duration activities which create minimal dust**  Include but not limited to:  e.g., Activities that require access to duct spaces, cutting of walls, ceilings, sanding of walls for painting, plumbing that requires disruption to water supply of more than one patient care area (> two rooms) for less than 30 minutes. |
| **Type C** | **Work that generates a moderate to high level of dust or requires demolition or removal of any fixed building components or assemblies.**  Include but not limited to:   - Sanding of walls for painting or wall covering - Removal of floor coverings, ceiling tiles and case work - New wall construction - Minor duct work or electrical work above ceilings - Major cabling activity - Any activity that cannot be completed within a single work shift. |
| **Type D** | **Major demolition, construction & renovation projects**  Includes but not limited to:   - Activities that require consecutive work shifts - Require heavy demolition or removal of a complete cabling system - New construction / new building project. |

**STEP 2:** Using the following table, identify the patient risk groups affected by the activity. If more than one risk group will be affected, select the higher risk group.

**RISK GROUPS. IDENTIFY PATIENT AT RISK (circle area involved):**

| **LOW RISK** | **MEDIUM RISK** | **HIGH RISK** | **HIGHER RISK** |
| --- | --- | --- | --- |
| - All office area - Nonclinical areas | - Admitting Unit - Outpatient Areas - Food prep areas - Radiology - Nuclear Medicine - MRI - Endoscopy Unit - Outpatient Physical Therapy (Rehab) - Psychiatric Services (outpatient) - Cardiology services (outpatient) | - Trauma & Emergency Department - Labor & Delivery Ward - Pediatrics Wards - Pharmacy - Newborn Nursery - Clinical Pathology - Day Care Surgery - Central Stores - Laboratories - Medical Units - Surgical Units - Hemodialysis Unit | - Bone Marrow Transplant Unit - Burn Intensive Care Unit - Cardiac Cath Lab - Pharmacy Sterile Unit - Operating Rooms - Negative Air / Positive Air Pressure Rooms - Isolation Rooms (in all wards/ Units) - Intensive Care Units - Cardiac Intensive Care Unit - Dialysis Unit - PICU - CSSD - Oncology Ward - Any area / ward / unit caring for immunocompromised patients |

**STEP 3: Match the** planned **Construction activity type** (A, B, C, D) with the **Patient Risk Group** (low, medium, high, highest) to determine the **Class of Precautions** (I, II, III, IV or level of IPC activities required.

**RISK CLASS DETERMINATION**

| **PATIENT RISK GROUPS** | **CONSTRUCTION**  **ACTIVITY**  **TYPE A** | **CONSTRUCTION**  **ACTIVITY**  **TYPE B** | **CONSTRUCTION**  **ACTIVITY**  **TYPE C** | **CONSTRUCTION**  **ACTIVITY**  **TYPE D** |
| --- | --- | --- | --- | --- |
| Low Risk | I | II | II | III / IV |
| Medium Risk | I | II | III or II | IV |
| High Risk | I | II or III | III / IV | IV |
| Highest Risk | II / III | III / IV | III / IV | IV |

**Appendix B****: Water Management Construction (WMC)- ICRA**

**Pre-Construction Risk Assessment Checklist**

Use this checklist in conjunction with WMC ICRA Checklist for improving water quality and safety associated with construction activities. Please identify all risk factors within the construction project scope. This checklist should be reviewed by IPC and Facilities Management to determine WMC Project Category, Building Occupant Risk Groups, and Risk Mitigation Level. Describe additional information in the Note(s) Section to clarify extend of construction project scope.

| Project Name: | | | | | Facility: | | |
| --- | --- | --- | --- | --- | --- | --- | --- |
| Project Scope of Work Description: | | | | | | | |
| Location/Department: | | | | | Project date: | | |
| 1. **External construction or projects (outside main buildings)** | | | | | | | |
| **SITE WMC RISK FACTORS** | | | | **YES** | **NO** | | **NOTES SECTION** |
| **Excavation** | | | | | | | |
| - Potential for soil and sediment invasion | | | |  |  | |  |
| - Describe location(s) | | | |  |  | |  |
| **Underground utility connections** | | | | | | | |
| - Potential for soil and sediment invasion | | | |  |  | |  |
| **Repressurization of building main / point-of-entry water system** | | | | | | | |
| - Will the building main entry be shut-down or experience re-pressurization? | | | |  |  | |  |
| **Site / Civil Water service disruption** | | | | | | | |
| - New construction tie-ins | | | |  |  | |  |
| - Replacement valves | | | |  |  | |  |
| - Hydrants | | | |  |  | |  |
| - Meters | | | |  |  | |  |
| - Pumping failures | | | |  |  | |  |
| - Pipeline breaks | | | |  |  | |  |
| - Other system repairs | | | |  |  | |  |
| - Emergency conditions | | | |  |  | |  |
| **Lengthy underground piping connections** | | | | | | | |
| - Site routing of water utility piping | | | |  |  | |  |
| - Fire hydrant locations and piping routing with dead-ends | | | |  |  | |  |
| - Distance from building main connection to the street connection / invert | | | |  |  | |  |
| **Vibration activities** | | | | | | | |
| - Pile Driving / structural foundation | | | |  |  | |  |
| - Jackhammering | | | |  |  | |  |
| - Saw cutting | | | |  |  | |  |
| **Demolition activities** | | | | | | | |
| - Creates air fumes of dust or water aerosols toward patient care areas | | | |  | |  |  |
| - Drift of debris toward cooling towers | | | |  | |  |  |
| - Drift of debris toward cooling HVAC intake vents | | | |  | |  |  |
| **Demolishing building water system components** | | | | | | | |
| - Impacting other building water supply connection points | | | |  | |  |  |
| - Other Site / Civil utility demolition activities | | | |  | |  |  |
| **Demolishing underground tunnels** | | | | | | | |
| - Utility | | | |  | |  |  |
| - Walking / passage / transportation | | | |  | |  |  |
| **Construction equipped with water reservoirs (i.e. typically spray activities)** | | | | | | | |
| - Water tankers | | | |  | |  |  |
| - Paving equipment | | | |  | |  |  |
| - Spray nozzles | | | |  | |  |  |
| - Misters | | | |  | |  |  |
| - Other | | | |  | |  |  |
| **Water main disruptions** | | | | | | | |
| - Opportunity for water main breakage | | | |  | |  |  |
| - Length of shut down in hours / days | | | |  | |  |  |
| - Off-site construction of PUB water delivery system | | | |  | |  |  |
| **Central Utility Plant Modifications / Alterations** | | | | | | | |
| - Underground utility connections | | | |  | |  |  |
| **Cooling Towers** | | | | | | | |
| - Replacement | | | |  | |  |  |
| - Addition | | | |  | |  |  |
| **Disinfection of underground utility connections or building** | | | | | | | |
| - Does the project call for building water main disinfection? | | | |  | |  |  |
| - When during the project is this activity scheduled to be performed? | | | |  | |  |  |
| **High Water Age / Stagnation Challenges (circle one)** | | | | | | | |
| How long will building water system experience dormancy or shut-downs? | | | |  | |  |  |
| <1 day | < 7 days | < 30 days | > 30 days |  | |  |  |
| **Inadequate residual disinfectant** | | | | | | | |
| Is disinfectant residual measurement between total residual oxidant (TRO) > 0.5 ppm or < 4.0 ppm / or / free residual oxidant (FRO) ≥ 0.2 ppm | | | |  | |  |  |
| **Provide verification of existing residual disinfectant measurements** | | | | | | | |
| - Incoming PUB water main | | | |  | |  |  |
| - Pre – Post water softener | | | |  | |  |  |
| - Return hot water loop system | | | |  | |  |  |
| - Distal distribution points on each floor of construction | | | |  | |  |  |
| **Confirm existing temperature control ranges** | | | | | | | |
| - Hot water storage temperature | | | |  | |  |  |
| - Hot water range (per WMP at fixture delivery) | | | |  | |  |  |
| - Cold water range (per WMP) | | | |  | |  |  |
| - Does the project utilize point-of-use mixing valves? | | | |  | |  |  |
| **Unoccupied areas or low or no use areas pre or post occupancy** | | | | | | | |
| - Shell areas with water in piping system | | | |  | |  |  |
| - Unoccupied areas with water in piping system | | | |  | |  |  |
| - Low use areas with water in piping system | | | |  | |  |  |
| 1. **Internal construction or projects (within main buildings)** | | | | | | | |
| **Vibration activities** | | | | | | | |
| - Demolition | | | |  | |  |  |
| - Jackhammering | | | |  | |  |  |
| - Saw cutting | | | |  | |  |  |
| - What departments are above, below, or downstream / near vibration activities? | | | |  | |  |  |
| **Efficiency design challenges** | | | | | | | |
| - Water system design for conservation measures | | | |  | |  |  |
| - Participating in locally recognized building rating systems, e.g., Green Mark, WELS rating, where applicable | | | |  | |  |  |
| - Auto-fixtures (electronic, sensor, or push button e.g., surgical scrub sinks) | | | |  | |  |  |
| - Aerators | | | |  | |  |  |
| - Ligature-resistant fixtures (i.e., behavioral health, security fixtures) | | | |  | |  |  |
| - Other – mixed temperature fixtures, etc. | | | |  | |  |  |
| **Re-pressurization (start-up and shut-down)** | | | | | | | |
| - Will any part of the building water system experience re-pressurization? | | | |  | |  |  |
| **Building / Plumbing Water service disruption** | | | | | | | |
| - New construction tie-ins | | | |  | |  |  |
| - Replacement valves | | | |  | |  |  |
| - Meters | | | |  | |  |  |
| - Pumping failures | | | |  | |  |  |
| - Other system repairs or component replacement | | | |  | |  |  |
| - Emergency conditions | | | |  | |  |  |
| **Construction equipment with water reservoirs typically with spray activities** | | | | | | | |
| - Showers | | | |  | |  |  |
| - Spray nozzles | | | |  | |  |  |
| - Misters | | | |  | |  |  |
| - other | | | |  | |  |  |
| **Disinfection of BWDS** | | | | | | | |
| - Does the project call for BWDS disinfection? | | | |  | |  |  |
| - Have ports and isolation valves been installed for this section of the building? | | | |  | |  |  |
| - When during the project is this activity scheduled to be performed? | | | |  | |  |  |
| **High Water Age / Stagnation Challenges (circle one)** | | | | | | | |
| How long will building water system experience dormancy or shut-downs? | | | |  | |  |  |
| <1 day | < 7 days | < 30 days | > 30 days |  | |  |  |
| **Inadequate residual disinfectant** | | | | | | | |
| Is disinfectant residual measurement between total residual oxidant (TRO) > 0.5 ppm or < 4.0 ppm / or / free residual oxidant (FRO) ≥ 0.2 ppm | | | |  | |  |  |
| **Provide verification of existing residual disinfectant measurements** | | | | | | | |
| - Incoming PUB water main | | | |  | |  |  |
| - Pre – Post water softener | | | |  | |  |  |
| - Return hot water loop system | | | |  | |  |  |
| - Distal distribution points on each floor of construction | | | |  | |  |  |
| **Confirm existing temperature control ranges** | | | | | | | |
| - Hot water storage temperature | | | |  | |  |  |
| - Hot water range (per WMP at fixture delivery) | | | |  | |  |  |
| - Cold water range (per WMP) | | | |  | |  |  |
| - Does the project utilize point-of-use mixing valves? | | | |  | |  |  |
| **Unoccupied areas or low or no use areas pre or post occupancy** | | | | | | | |
| - Shell areas with water in piping system | | | |  | |  |  |
| - Unoccupied areas with water in piping system | | | |  | |  |  |
| - Low use areas with water in piping system | | | |  | |  |  |
| **Central Utility System Modifications / Alterations** | | | | | | | |
| - Water heaters | | | |  | |  |  |
| - Heat exchanges | | | |  | |  |  |
| - Water storage | | | |  | |  |  |
| - Hot water loop system | | | |  | |  |  |
| - Boiler system | | | |  | |  |  |
| - Other central building water system compounds | | | |  | |  |  |

| **Reference:** | Scanlon MM, Gordon JL, Tonozzi AA, and Griffin SC (2022). Reducing the Risk of Healthcare Associated Infections from *Legionella* and Other Waterborne Pathogens Using a Water Management for Construction (WMC) Infection Control Risk Assessment (ICRA) Tool. *Infectious Disease Reports*, 14(3). |
| --- | --- |

**Appendix C****: Water Management Construction ICRA Checklist**

Step 1.

Using Table 1, evaluate the Building Water Distribution System (BWDS) construction activities and scope of work to be performed, duration and level of water age for the project, and determine the Water Management Construction (WMC) Project Category:

**Table 1: WMC Project Category**

| **Category** | **BWDS Construction Activities and Scope of Work** | **Water Age Category** |
| --- | --- | --- |
| **A** | - Minimally invasive BWDS - Brief duration | Low (< 24 hours) |
| **B** | - Small scale BWDS - Short duration | Modest (< 7 days) |
| **C** | - Moderate to high levels of BWDS construction | Medium (< 30 days) |
| **D** | - Major BWDS demolition, renovation, infrastructure, and/or new construction | High (> 30 days) |

| **Category** | **Examples:** |
| --- | --- |
| **A** | **BWDS inspection, maintenance/repair and non-invasive activities of brief duration, and low water age.**  Includes but not limited to:   - replacing fixture trim(s) - replacing fixture “in-kind” (i.e., meaning 1:1 or like for like) - impact and risk is only to building users in the immediate area of construction - water by fixture or area is shut down for < 24 hours (minimal water age/stagnation) |
| **B** | **Small scale BWDS, short duration activities which create minimal water disruption, and modest water age.**  Includes but not limited to:   - replacing or installing fixtures and trim - working within wall cavities and / or ceiling areas - water by fixture or area is shut down for < 7 calendar days (1 work week for water age) |
| **C** | **Work generates moderate to high BWDS disruption or removal of any fixed BWDS components or assemblies with medium water age.**  Includes but not limited to:   - plumbing work requiring multiple fixtures (existing, replacement or new) - major water system component replacement (boilers, heaters, water main, etc.) - work in wall cavities or ceilings with major disruption to local and downstream occupied areas - change of functional building space programme (i.e., moving / changing room or dept. functions) in existing building - water by fixture, component, or area is shut down < 30 days |
| **D** | **Major BWDS demolition, renovation, infrastructure, and/or new construction projects with high water age.**  Includes but not limited to:   - change in functional building space programme (i.e., series of rooms and departments) - tenant improvements (i.e., existing buildings, or tenant space within unoccupied buildings) - new shell and core buildings, additions, or expansions on campus (i.e., near existing patient environments) - acquisition of building with unknown water quality / safety conditions - infrastructure projects connecting to building water systems (i.e., underground piping, utility tunnels, etc.) - water by fixture or area is not active (new start-up) or was shut down (> 30 days) |

**Step 2.**

Using Table 2, identify the Occupant Risk Groups (i.e., patients, visitors, volunteers, staff, etc.) and affected departmental areas. If more than one building occupant risk groups will be affected, select the higher risk group. Contact Facilities Management or IPC Department if any risk group needs further clarification for relationships to the BWDS construction scope of work.

**Table 2: Building Occupant Risk Groups**

| **Low Risk** | **Medium Risk** | High Risk | Highest Risk |
| --- | --- | --- | --- |
| - Office areas - Non-patient areas | - Cardiology - Nuclear Medicine - Physiotherapy / Occupational Therapy / Speech Therapy Department - Radiology/MRI - Patient care areas not covered under high or highest risk groups - Public corridors (through which patients, supplies and linen pass) - Lab not specified as high or highest risk groups - Cafeteria / Kitchen - Material management department - Linen room - Pharmacy | - Coronary Care Unit - Emergency Medicine - Labour & Delivery - Laboratories (specimen) - Newborn Nursery - Endoscopy Centre - Paediatrics - Pharmacy laboratory - Medical and Surgical wards (including HD and ICA) - Rehabilitation ward - Vascular and interventional radiology | - Any areas caring for immuno-compromised patients - Oncology ward - Bone marrow transplant unit - Haematology ward and Centre - Neonatal ward - Burn Unit - Cardiac Cath Lab / angiograph procedure areas - Central Sterile Supply - Intensive Care Units - Operating theatres including Ambulatory Surgery - Dialysis Centre |

**Step 3.**

Match the Building Occupant Risk Group (Low, Medium, High, Highest) with the planned WMC Project Category (A, B, C, D) on the WMC Infection Control Risk Assessment Matrix (Table 3) to determine the WMC Risk Mitigation Level (WMC - 1, 2, 3, or 4) for hazard control strategies to be implemented over the entire duration of the construction project scope (Table 4).

Larger scale projects (WMC - 4 with > 30 days of dormancy or new start-up) as indicated in WMC-ICRA Category D should conduct a pre-construction risk assessment (PCRA). The PCRA checklist should be reviewed by IPC and Project Officer.

**Table 3: WMC Infection Control Risk Assessment Matrix**

| **Occupant Risk Group** | **WMC Project Category** | | | |
| --- | --- | --- | --- | --- |
|  | **A** | **B** | **C** | **D** |
|  | Minimally invasive BWDS, brief duration, and low water age (< 24 hours) | Small scale BWDS, short duration, and modest water age (< 7 days) | Moderate to high levels of BWDS construction, and medium water age (< 30 days) | Major BWDS demolition, renovation, infrastructure, and/or new construction with high water age (>30 days) |
| **Low Risk** | **WMC - 1** | **WMC - 2** | **WMC - 3** | **WMC - 3 / 4** |
| **Medium Risk** | **WMC - 1** | **WMC - 2** | **WMC - 3** | **WMC - 4** |
| **High Risk** | **WMC - 2** | **WMC - 3** | **WMC- 3 / 4** | **WMC - 4** |
| **Highest Risk** | **WMC - 2** | **WMC - 3 / 4** | **WMC - 3 / 4** | **WMC - 4** |

BWDS: building water distribution system

**Step 4.**

Review, finalize, and implement the selected WMC Risk Mitigation Levels determined as risk group (Table 4). All mitigation measures (hazard controls) and associated numeric values (i.e., temperature, residual free chlorine levels, pH, or other) need to be reviewed, coordinated and implemented in context with the organization’s on-going Water Management Programme. Contact Facilities Management or IPC Department for clarification on individual hazard controls defined for the project duration.

**Table 4: WMC Risk Mitigation Level and Hazard Control Strategies**

| ***WMC – 1*** | 1. Flush fixture (hot) for minimum 4 minutes; following flushing collect water temperature using digital thermometer; perform the same minimum 4-minutes flushing (cold) and collect water temperature; record both measurements. 2. Perform repair or replacement of plumbing components (i.e., plumbing fixture, trim or other). 3. When construction activities are completed, and area is ready to return to service, flush the fixture for minimum of 4 minutes hot, then 4 minutes cold. Take corresponding temperature and residual free chlorine levels measurements. Repeat steps until measurements are the same or better than pre-existing conditions:  - Outlet temperature: hot water range (45^0^C to 48.9^0^C for new building; maximum 40 ^0^C for existing buildings). - Residual free chlorine levels: minimal of 0.20 ppm  1. Report any odour, discoloured water, flecks or floating debris at baseline or at work completion; none should be present. 2. Record information on organization’s flushing form or in the project information record system. |
| --- | --- |

| **WMC – 2** | **Perform ALL of WMC – 1 risk mitigation and adjust for scale of project and**:   1. For high and highest risk areas:    - Prior to construction activities determine baseline measurements (temperature, residual free chlorine levels, pH, or other).    - Collect residual free chlorine levels using a digital colorimeter instrument and record measurement. 2. Establish enclosure to prevent aerosolized water (and potential pathogens) from dispersing into the environment. 3. Close door of area (i.e., patient room door, toilet/shower room door, etc.) 4. Install non-flammable visqueen or clear plastic sheeting or other approved vapor barrier for protection 5. Install isolation valve, backflow prevention device, or other piping isolation method, when needed 6. Construction staff to:  - leave barriers in place until all plumbing work is complete including flushing activities - thoroughly clean and dry area(s) upon completion of construction work - remove barriers or seals in place  1. Environmental Services to perform routine cleaning before the area is occupied. 2. When construction activities are completed, and area is ready to return to service, flush the fixture for minimum of 4 minutes hot, then 4 minutes cold. Take corresponding temperature and residual free chlorine levels measurements. Repeat steps until measurements are the same or better than pre-existing conditions: 3. Outlet temperature: hot water range (45^0^C to 48.9^0^C for new building; maximum 40 ^0^C for existing buildings).  - Residual free chlorine levels: minimal of 0.20 ppm  1. For high and highest risk areas:    - Carry out analytical laboratory sampling for water quality (drinking water standard) when necessary (depends on the work carried out), residual free chlorine levels and culture for *Legionella* sp. for water outlets of renovated cubicle OR the 3 designated sites for sampling along a floor. |
| --- | --- |
| **WMC – 3** | **Perform ALL of WMC – 1 and 2 risk mitigation levels and adjust for scale of project** **and:**   1. Prior to construction activities determine baseline measurements (temperature, residual free chlorine levels, pH, or other). 2. Collect residual free chlorine levels using a digital colorimeter instrument and record measurement. 3. Perform flushing protocol (4 min. per day every other day @ all fixtures in *unoccupied* areas adjacent to the construction zone). Report on flushing form (or record in the project information record system). 4. Obtain residual free chlorine levels and temperature readings post flushing activities 1 day per week in unoccupied and occupied areas at 10 % of designated fixture locations as representative sample of fixtures to maintain adequate temperature and residual free chlorine levels where there is no valve to isolate the water system from renovated area. Report on fixture analysis form (or record in the project information record system). 5. Review any disinfection (i.e., hyperchlorination) procedures to be performed with the Owner’s Project Representative including location(s), method, schedule, and timing to return water system for potable usage, where applicable. Provide any reports of activities for building water main (i.e., point-of-entry), building distribution systems (hot and / or cold). 6. Where necessary (where there is no valve to isolate the water system from renovated area), provide any temporary inline or point-of-use filtration during construction for designated sinks, showers, or other fixtures or piping lines to reduce risk of exposure. 7. Review installation for patient and medical equipment with water reservoirs (i.e., ice machines or other) on the project and preventive maintenance prior to occupant start up. 8. Carry out analytical laboratory sampling for water quality (drinking water standard) when necessary (depends on the work carried out), free chlorine levels and culture for *Legionella* sp. for water outlets of renovated cubicle OR the 3 designated sites for sampling along a floor. |
| **WMC - 4** | **Use WMC – 1, 2, and 3 risk mitigation levels and prepare a project specific WMC plan and operations and**:   1. Contact the Building Owner’s Project Representative for preparing a WMC Project Analysis. 2. Conduct a project-specific pre-construction risk assessment for potential growth and spread of waterborne pathogens. Complete WMC-ICRA Pre-Construction Risk Assessment Checklist.  - Review site / construction activity risk factors - Review building design and construction risk activity risk factors  1. Based upon the risk assessment prepare a project-specific WMC plan for commissioning the building water system(s) per recommendations by the hospital Water Management Committee.  - Establish a WMC plan with scheduled milestones starting from the date of water activation through first-day of patient care operations - Implement / operationalize project specific controls (i.e., protocols for flushing, temperature, and residual free chlorine levels) - Confirm WMC plan & operations with verification and validation.  1. Obtain Building Owner’s Project Representative approval of the WMC plan, process, and documentation. 2. Implement the agreed upon WMC Plan for achieving water quality and safety. 3. Obtain approval from the hospital Water Management Committee for new start-up before initiating patient care operations. |
